# Association of serum 25-hydroxy-vitamin D concentration and risk of mortality in cancer survivors in the United States

**DOI:** 10.1186/s12885-024-12304-8

**Published:** 2024-04-30

**Authors:** Xiaofei Mo, Chen He, Fengfeng Han, Hui Yan, Xueqin Chen, Yuetao Wang, Mingge Zhou

**Affiliations:** 1 Department of Nuclear Medicine, The Third Affiliated Hospital of Soochow University, Changzhou, 213003 Jiangsu China; 2 Changzhou Key Laboratory of Molecular Imaging, Changzhou, 213003 Jiangsu China

**Keywords:** Cancer diagnosis, Vitamin D, NHANES, Mortality, Risk

## Abstract

**Purpose:**

Cancer survivors have a high risk of mortality, and vitamin D (VD) is associated with the risk of mortality. This study is aim to examine the impact of VD on mortality in cancer survivors.

**Methods:**

A prospective study was conducted using data from the National Health and Nutrition Examination Survey. Participants were obtained information on their baseline characteristics, dietary habits, comorbidities, lifestyle, and serum 25-hydroxy VD [25(OH)D] concentrations. The weighted Cox proportional hazard and competing risk regression models were used to estimate the hazard ratio and 95% confidence intervals (HR, 95% CI) of mortality for different serum 25(OH)D concentrations. Restricted cubic spline (RCS) curves were utilized to illustrate the dose–response relationship between serum 25(OH)D concentrations and mortality.

**Results:**

The study encompassed 2,495 participants with cancer diagnoses. Multivariate models indicated that, compared to serum 25(OH)D concentrations below 58.5 nmol/L, concentrations exceeding 81.6 nmol/L were associated with reduced HRs for all-cause mortality (HR = 0.70; 95% CI: 0.56–0.87), cardiovascular mortality (HR = 0.53; 95% CI: 0.32–0.86), and cancer-specific mortality (HR = 0.66; 95% CI: 0.45–0.99). RCS curves revealed “L-shaped” associations between serum 25(OH)D concentration and both all-cause and cancer-specific mortality, with threshold effects at 87.9 nmol/L and 84.6 nmol/L, respectively. Conversely, the relationship between serum 25(OH)D concentration and cardiovascular mortality exhibited a more linear pattern, with a threshold at 88.7 nmol/L. Subgroup analyses highlighted a gender-specific interaction that elevated serum 25(OH)D concentrations were significantly more protective against mortality in males than in females, especially regarding cancer-specific mortality (*P*-interaction = 0.009).

**Conclusion:**

Elevated serum 25(OH)D concentrations were correlated with decreased risks of all-cause, cardiovascular, and cancer-specific mortality in cancer survivors, with benefit thresholds at 87.9, 88.7, and 84.6 nmol/L, respectively. These findings suggested that cancer survivors might benefit from higher vitamin D recommendations than the general population.

**Supplementary Information:**

The online version contains supplementary material available at 10.1186/s12885-024-12304-8.

## Background

Vitamin D (VD) is a significant nutrient that impacts human health. Extensive researches have focus on the associations between VD and a range of disease conditions, including cardiovascular diseases [1], inflammation responses [2], and cancer development [3]. These studies commonly indicate that a deficiency in VD can increase the risk of cardiovascular-related mortality [4], contribute to the onset of inflammatory autoimmune diseases [5], and exacerbate metabolic disorders such as hypertension [6] and type 2 diabetes [7]. Moreover, VD deficiency is predominated in the world [8], many health organizations have advised it is necessary to monitor people’s VD status in different area and risk stratification [9], in order to establish a valid recommend value of VD status in body that decrease related risks.

Recognizing the critical role of VD, it is imperative to determine an optimal serum concentration that reduce the risk of diseases development and mortality. It is well-established that 25-hydroxy-VD [25(OH)D] serves as a reliable biomarker for assessing VD status in the body because of its stability and prolonged half-life in the bloodstream [10]. Serum 25(OH)D, the principal storage form of VD and direct precursor to its active form [4], has been identified the thresholds associated with the lowest mortality risk in patients with conditions like hypertension [11], diabetes [12], and hyperlipidemia [13] in several studies. While previous studies have explored the impact of VD supplementation on mortality in cancer patients [14, 15], the findings have been inconsistent [14]. Considering the variability in individuals’ baseline VD status, dietary practices, sun exposure, and absorption and conversion efficiency of VD, evaluating the effect based solely on the dosage of VD supplements is inadequate. Therefore, a consensus has emerged recommending the use of circulating 25(OH)D concentration to identify individuals with VD deficiency and to guide the design of randomized clinical trials [16]. Moreover, as cancer survivors face a heightened mortality risk compared to the broader population, the optimal serum 25(OH)D concentrations may diverge from the current standards, making it vital to establish a specific target concentration for this demographic.

This study utilizes data from the National Health and Nutrition Examination Survey (NHANES) to conduct a prospective analysis exploring the link between serum 25(OH)D concentrations and the risk of all-cause, cardiovascular, and cancer-specific mortalities in participants with a cancer diagnosis. Additionally, this research seeks to identify an optimal serum 25(OH)D threshold to guide prognostic management in these patients.

## Methods

### Study population

This study utilized data from the NHANES, an ongoing national survey orchestrated by the National Center for Health Statistics to appraise the health and nutritional status of Americans across all ages [17]. NHANES comprehensively gathers data relevant to demographics, socioeconomic status, diet, health examinations, medical evaluations, laboratory analyses, and participant questionnaires. Participation was contingent upon obtaining written informed consent from all subjects.

The study cohort consisted of individuals aged between 20 and 80 who had completed the NHANES interview, examination, and the dual 24-h dietary recall process across the 2003–2016 cycles. Mortality status was determined using the Public-Use Linked Mortality File, updated until December 31, 2019. This database connects NHANES participants to the National Death Index, with individual records linked via the Sequence Number (SEQN) provided by the National Center for Health Statistics. Mortality was classified based on the International Classification of Diseases, Tenth Revision (ICD-10); with cardiovascular mortality encompassing heart diseases (ICD-10 codes I00-I09, I11, I13, I20-I51) and cerebrovascular diseases (ICD-10 codes I60-I69), and cancer mortality including deaths from malignant neoplasms (ICD-10 codes C00-C97). Follow-up commenced upon completion of participation at the Mobile Examination Center (MEC) and continued until the cutoff date (December 31, 2019), and person-time calculated from serum 25(OH)D examination to either date of death or end of follow-up. The medical conditions section of the questionnaire, modeled after the U.S. National Health Interview Survey, collected cancer history, detailing type of cancer, age at diagnosis, and occurrences of any secondary cancer.

**Fig. 1:**
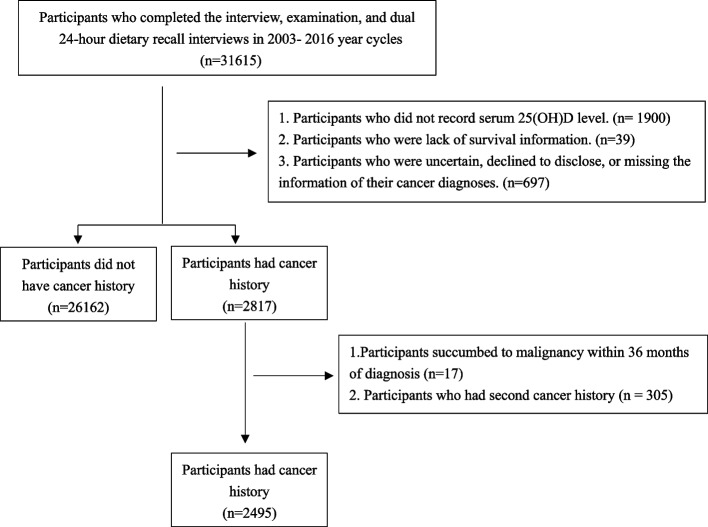
The flow chart of participants enrollment

There were 36165 participants completed NHANES interview and MEC examination. After excluding participants lacking serum 25(OH)D concentrations (*n* = 1900), those without survival data (*n* = 39), and individuals who were uncertain, declined to disclose, or missing the information of their cancer diagnoses (*n* = 697), the study cohort comprised 26,162 participants with no history of cancer and 2,817 who had been diagnosed with the disease. Subsequently, given that various cancer stages and treatment approaches can significantly affect the life expectancy of individuals with cancer, our analysis excluded participants who succumbed to malignancy within 36 months of diagnosis (*n* = 17), as these cases may represent advanced-stage type or ineffective treatment that could distort overall survival outcomes. Moreover, participants who have diagnosed with a second cancer were also excluded (*n* = 305), as they might have worse survival expectancy that could generate bias in the analysis. The flow chat of participants enrollment is showed in Fig. 1.

### Measurement assay of Serum 25(OH)D

In this study, serum 25(OH)D encompasses 25(OH)D2, 25(OH)D3, and epi-25(OH)D3. The quantification was performed using ultra-high-performance liquid chromatography-tandem mass spectrometry (UHPLC-MS) [18], and the units are expressed as nanomoles per liter (nmol/L). Detailed methodological procedures are available in the NHANES data documentation (https://wwwn.cdc.gov/nchs/data/nhanes/2017-2018/labmethods/VID-J-MET-508.pdf) and analytical note (https://wwwn.cdc.gov/nchs/nhanes/vitamind/analyticalnote.aspx).

### Statistical analysis

The present study utilized various covariates obtained from enrolled participants, which included: (1) Demographic information such as age, ethnicity (classified into Non-Hispanic White, Non-Hispanic Black, Mexican American, and Other), gender (male or female), and marital status (married/living with partner or never married/separated/widowed/divorced); (2) Physical examination data: Body Mass Index (BMI), calculated as weight in kilograms over height in meters squared and stratified into normal weight (< 25), overweight (25 – 29.9), or obese (≥ 30.0) [19]; and blood collection timing, differentiated by “November 1 through April 30” and “May 1 through October 31”; (3) Lifestyle factors: smoking status (current, never, or former smoker), alcohol consumption (nondrinkers, moderate drinkers with < 2 drinks/day for males and < 1 drink/day for females, and heavy drinkers with ≥ 2 drinks/day for males and ≥ 1 drink/day for females, and not recorded) [20], dietary quality assessed by the Healthy Eating Index-2015 and classified into poor (< 50.0), needs improvement (50.0 – 79.9), or healthy (≥ 80.0) diet quality [21]; and physical activity levels expressed in metabolic equivalent (MET) minutes per week, categorized by the International Physical Activity Questionnaire [22] and the World Health Organization recommendations as low (no activity), moderate (< 600 MET-min/week), or high (≥ 600 MET-min/week) [23]; (4) Health-related information: comorbidity levels determined using self-reported questionnaires and examinations and graded using the Charlson comorbidity index (CCI) [24] (scores of 0–2, 3, or > 3); medication usage within the last 30 days (yes or no); (5) Cancer types, primarily breast, prostate, non-melanoma skin, and other, predicated on their prevalence among the cohort; and time since cancer diagnosis, categorized into ≤ 5 years, > 5 years, or not recorded.

Sample weights, strata, and primary sampling units were used to account for the complex survey design according to the NHANES analytic guidelines [25]. Participants were categorized into tertiles according to their serum 25(OH)D concentrations. Continuous variables underwent normality testing, and the characteristics of the study population were depicted as means (± SE) for continuous variables and as percentages for categorical variables. We assessed the association of continuous variables using analysis of variance (ANOVA) and compared categorical variables via the Chi-square test. Spearman’s correlation coefficients were determined for all variables, and multicollinearity was checked using the variance inflation factor (VIF), with values below 10 denoting the absence of multicollinearity. Detailed results of Spearman’s correlations and VIF for each variable can be found in the Supplementary materials.

We utilized weighted Cox proportional hazards (COXPH) models for survival analysis to explore the relationship between 25(OH)D concentrations and all-cause mortality, confirming the proportional hazards assumption with Schoenfeld residuals (Supplementary Materials). Weighted competing risks regression (CRR) models were used to estimate the association of serum 25(OH)D concentrations with cardiovascular and cancer-specific mortality. The multivariate models included adjustments for age, gender, ethnicity, marital status, and the time of blood draw in the initial model, followed by additional adjustments for body mass index (BMI), Charlson Comorbidity Index (CCI), Healthy Eating Index (HEI), medication intake, smoking status, alcohol consumption, physical activity levels, cancer types, and the time since cancer diagnosis. A test for a linear trend across the tertiles of serum 25(OH)D was conducted by designating median values to each tertile and considering it a continuous variable. Interaction and stratified analyses were conducted on the basis of gender (male and female) and ethnicity (non-Hispanic White and other ethnicities). Additionally, as part of a sensitivity analysis, serum 25(OH)D concentrations were categorized into deficiency (< 50 nmol/L), insufficiency (50—75 nmol/L), and sufficiency (> 75 nmol/L) status [26] to perform survival analyses using the aforementioned procedure.

For the dose–response relationship between serum 25(OH)D concentrations and cause-specific mortalities, we employed restricted cubic spline (RCS) models with three knots at the 10th, 50th, and 90th percentiles. These models accounted for a range of covariates, including age, gender, ethnicity, marital status, time of blood draw, BMI, CCI, medication intake, smoking status, alcohol consumption, physical activity levels, cancer types, and the duration since cancer diagnosis. We considered a two-sided *p*-value of less than 0.05 to be statistically significant. The analyses were conducted using version 4.2.1 of the R software, incorporating packages such as ‘rms’, ‘cmprsk’, ‘survminer’, ‘svycoxph’, and ‘plotRCS’ for modeling and ‘corrplot’ for the visualization of correlations.

## Results

### Characteristics of the participants

As Fig. 1 shown, there were 2495 participants meeting the defined criteria that included in our study. We divided participants into three groups at 33% and 67% percentiles of serum 25(OH)D concentration distribution, with the values of 58.1 and 81.6 nmol/L. The characteristics of the included participants were summarized in Table 1. The mean age of participants was 62.10 ± 0.38 years. A majority of 57.4% were female, and 87.8% identified as Non-Hispanic White, followed by 5.0% as Non-Hispanic Black, 2.1% as Mexican American, and 5.1% as Other Races. Regarding 25(OH)D concentrations, 20.8% (*n* = 518) of the participants were classified as vitamin D deficient (< 50 nmol/L), and 36.5% (*n* = 910) as insufficient (50—75 nmol/L) [27]. Analysis of participant characteristics by tertiles of 25(OH)D concentrations revealed that those in the highest tertile had the oldest average age (63.81 ± 0.63 years), the highest percentages of females (61.4%) and Non-Hispanic Whites (93.7%), and the greatest prevalence of a CCI score of 3 (36.6%). Conversely, these individuals had the lowest prevalence of obesity (26.6%), poor dietary quality (26.1%) as per the HEI-2015 index, in current smoking status (13.1%), and physical inactivity (17.7%). This group also exhibited the highest proportion of non-melanoma skin cancer (27.5%) and breast cancer (14.8%), yet the lowest proportion of prostate cancer (8.0%) and other types of cancer (49.7%). Participants in the middle tertile showed the highest proportion of males (47.1%) and those married or living with a partner (66.9%), but the lowest percentage with a CCI score greater than 3 (26.1%). This group also exhibited the highest proportion of prostate cancer (10.2%). Among those studied, 78.0% (*n* = 1,947) reported their age at cancer diagnosis, with an average interval between diagnosis and the end of follow-up of 230.08 ± 3.94 months. Participants who did not report their age at diagnosis had an average post-survey follow-up of 92.3 ± 2.10 months. Over 244,816 person-years of follow-up, a survival rate of 77.1% was observed, with 788 deaths comprising 348 (9.9%) from cardiovascular causes, 216 (6.3%) from malignancies, and 224 (6.7%) from other causes.

**Table 1:** The baseline of participants diagnosed with cancer according to serum 25(OH)D concentration

variable	Serum 25(OH)D concentration (nmol/mL)				
	Total	T1 (< 58.1)	T2 (58.1—81.4)	T3 (> 81.4)	*P*-value
Number	2495	831	842	822	
Age participated the survey	62.10(0.38)	60.28(0.68)	61.66(0.65)	63.81(0.63)	< 0.001
Gender					0.02
Female	1334(57.4)	452(57.4)	412(52.9)	470(61.4)	
Male	1161(42.6)	379(42.6)	430(47.1)	352(38.6)	
Ethnicity					< 0.001
Non-Hispanic White	1785(87.8)	466(77.6)	630(89.5)	689(93.7)	
Non-Hispanic Black	328( 5.0)	197(11.1)	71( 2.9)	60( 2.5)	
Mexican American	153( 2.1)	81(4.1)	55(2.1)	17(0.6)	
Others^a^	229( 5.1)	87(7.1)	86(5.5)	56(3.2)	
Marital status					0.002
Never married/separated/widowed/divorced	1031(36.2)	392(43.1)	313(33.1)	326(34.0)	
Married/living with partner	1464(63.8)	439(56.9)	529(66.9)	496(66.0)	
BMI					< 0.001
< 25 (normal weight)	699(29.2)	172(20.7)	239(28.6)	288(35.9)	
25 – 29.9 (overweight)	874(34.7)	252(30.2)	311(35.2)	311(37.5)	
≥ 30.0 (obesity)	922(36.1)	407(49.1)	292(36.1)	223(26.6)	
CCI					0.01
0—2	834(37.0)	303(39.9)	278(38.6)	253(33.5)	
3	831(33.5)	242(27.1)	294(35.3)	295(36.6)	
> 3	830(29.5)	286(33.1)	270(26.1)	274(29.9)	
HEI-2015					< 0.001
Poor dietary	829(32.9)	343(41.9)	272(33.3)	214(26.1)	
Need to improve	1570(63.3)	473(55.8)	539(63.8)	558(68.4)	
Healthy dietary	96( 3.7)	15(2.3)	31(2.9)	50(5.5)	
Smoke situation					< 0.001
Never	1132(45.1)	369(43.3)	389(46.7)	374(45.1)	
Now	376(16.2)	169(23.7)	107(13.4)	100(13.1)	
Former	987(38.7)	293(33.0)	346(39.9)	348(41.8)	
Alcohol					0.02
Never	945(31.3)	354(37.1)	305(28.8)	286(29.4)	
Mild to moderate	1257(55.9)	358(48.2)	457(59.7)	442(58.2)	
Heavy	217(10.4)	83(11.6)	65(10.0)	69( 9.9)	
Not record	76( 2.3)	36(3.1)	15(1.5)	25(2.5)	
Blood collection period					0.004
November 1 through April 30	1003(38.3)	407(45.3)	307(34.6)	289(36.5)	
May 1 through October 31	1492(61.7)	424(54.7)	535(65.4)	533(63.5)	
Physical acticity					< 0.001
No activity	531(22.4)	199(25.8)	193(24.9)	139(17.7)	
< 600 MET-minutes/week	1180(51.3)	319(41.1)	417(52.4)	444(57.8)	
≥ 600 MET-minutes/week	784(26.3)	313(33.1)	232(22.7)	239(24.6)	
Medication usage					0.01
Not use	345(15.4)	142(18.9)	119(16.0)	84(12.2)	
In use	2150(84.6)	689(81.1)	723(84.0)	738(87.8)	
Cancer type					< 0.001
Prostate	377( 8.8)	131( 8.1)	148(10.4)	98( 8.0)	
Breast	367(13.5)	112(12.3)	116(12.9)	139(14.8)	
Skin (non-melanoma)	436(23.4)	91(16.3)	165(24.6)	180(27.5)	
Other types	1315(54.3)	497(63.4)	413(52.0)	405(49.7)	
Age diagnosed with cancer					
Record [mean (SE)]	51.92(0.44)	49.59(0.72)	52.47(0.72)	53.06(0.76)	0.003
Not record (proportion)	548(21.4)	204(23.1)	173(21.2)	171(20.3)	0.5
Time since cancer diagnosis ^2^					
Mean (SE), months	116.47 (3.90)	111.96 (5.96)	109.37 (6.54)	128.12 (7.57)	0.12
> 5 years (proportion)	1895(97.83)	604(97.32)	654(98.18)	637(97.87)	0.68
Outcomes					< 0.001
Alive	1707(77.1)	529(70.8)	561(76.0)	617(82.6)	
Cardiovascular mortality	348( 9.9)	120(10.9)	137(11.2)	91( 8.2)	
Malignancies-specified mortality	216( 6.3)	78(8.7)	83(7.1)	55(4.0)	
Other causes	224( 6.7)	104(9.7)	61(5.8)	59(5.2)	

Measurement data were reported as means (standard error), and count data were presented as numbers (percentage). Means or percentages were adjusted for survey weights. Analysis of variance (ANOVA) was employed for continuous variables, while the Chi-square test was utilized for categorical variables to assess differences

^a^Other races include the Multi-Racial population and Hispanics

^b^Calculations were based only on participants with a recorded age of cancer diagnosis

### Association between serum 25(OH)D concentration with mortalities of participants with a cancer diagnosis

As Table 2 shown, a significant correlation was found between serum 25(OH)D levels and the risk of all-cause, cardiovascular, and cancer-specific mortality among participants with a cancer diagnosis. This association persisted after adjusting for multiple covariates. Specifically, for all-cause mortality, the hazard ratios (HRs) and 95% confidence intervals (CIs) for intermediate (58.1 – 81.6 nmol/L) and high (> 81.6 nmol/L) 25(OH)D concentrations were 0.85 (95% CI: 0.68 – 1.05) and 0.70 (95% CI: 0.56—0.87), respectively, as compared to low (< 58.1 nmol/L) concentrations, according to COXPH models. For cardiovascular mortality, the HRs for intermediate and high levels were 0.83 (0.54- 1.30) and 0.53 (0.32—0.86) respectively. In the context of cancer-specific mortality, the HRs for intermediate and high levels were 0.67 (95% CI: 0.40- 1.14) and 0.66 (95% CI: 0.45—0.99) respectively, as analyzed by CRR models. All models, except for those predicting cancer-specific mortality, demonstrated a linear trend between tertiles of serum 25(OH)D concentrations and HRs.

**Table 2:** The associations between different serum 25(OH) D concentrations with risk of all-cause, cardiovascular, and cancer-specific mortalities in participants with a cancer diagnosis

Outcomes	Serum 25(OH)D concentration (nmol/mL)			*P value for linear trend*
	T1 (< 58.1)	T2 (58.1—81.6)	T3 (> 81.6)	
All-cause mortality				
Model 1	1.00	**0.79 (0.65—0.98)**	**0.65 (0.52—0.80)**	< 0.001
Model 2	1.00	0.85 (0.68—1.05)	**0.70 (0.56—0.87)**	0.002
Cardiovascular mortality				
Model 1	1.00	0.76 (0.51—1.14)	**0.48 (0.30—0.75)**	0.002
Model 2	1.00	**0.83 (0.54—1.30)**	**0.53 (0.32—0.86)**	0.015
Cancer-specific mortality				
Model 1	1.00	**0.58 (0.36—0.96)**	**0.62 ( 0.41—0.93)**	0.022
Model 2	1.00	0.67 (0.40—1.14)	**0.66 ( 0.45—0.99)**	0.061

Bold font indicates p < 0.05

Model 1: Adjusted for age, race, gender, marital status, and timing of blood collection

Model 2: Includes adjustments from Model 1, with further consideration for Body Mass Index (BMI), Charlson Comorbidity Index (CCI), Healthy Eating Index (HEI), medication usage, smoking status, alcohol consumption, physical activity levels, types of cancer, and time since cancer diagnosis

Additionally, when serum 25(OH)D concentration was categorized into deficiency, insufficiency, and sufficiency, it was not found an obviously change on the result that highest risk was still confirmed in the lowest serum 25(OH)D concentration category. As presented in Table 3, deficient serum 25(OH)D concentrations were associated with increased HRs of 1.71 (95% CI: 1.32—2.21), 1.83 (95% CI: 1.14—2.95), and 2.02 (95% CI: 1.26—3.22) for all-cause, cardiovascular and cancer-specific mortality, respectively, in comparison to sufficiency levels.

**Table 3:** Associations of serum 25(OH)D concentrations classified as deficient, insufficient, or sufficient with the risk of all-cause, cardiovascular, and cancer-specific mortality among cancer survivors

Outcomes	Serum 25(OH)D concentration (nmol/mL)			*P for linear trend*
	Sufficiency (> 75)	Deficiency (< 50)	Insufficiency (50—75)	
All-cause mortality				
Model 1a	1.00	**1.96 (1.54—2.51)**	1.06 (0.87—1.29)	< 0.001
Model 2b	1.00	**1.71 (1.32—2.21)**	1.03 (0.84—1.25)	0.001
Cardiovascular mortality	1.00	**2.09 (1.30—3.35)**	1.23 (0.87—1.13)	0.006
	1.00	**1.83 (1.14—2.95)**	1.19 (0.84—1.69)	0.025
Cancer-specific mortality	1.00	**2.35 (1.55—3.54)**	0.88 (0.61—1.27)	0.003
	1.00	**2.02 (1.26—3.22)**	0.84 (0.59—1.22)	0.03

Bold font indicates *p *< 0.05

Model 1: Adjusted for age, race, gender, marital status, and timing of blood collection

Model 2: Includes adjustments from Model 1, with further consideration for Body Mass Index (BMI), Charlson Comorbidity Index (CCI), Healthy Eating Index (HEI), medication usage, smoking status, alcohol consumption, physical activity levels, types of cancer, and time since cancer diagnosis

In the subgroup analysis, HRs for cause-specific mortality still remained similar trend except some variations. According to Table 4, higher serum 25(OH)D concentrations were notably more protective against cause-specific mortality in males than in females. This distinction was particularly marked in the context of cancer-specific mortality, with a discernible interaction effect between serum 25(OH)D concentrations and gender. Male participants with serum 25(OH)D concentrations ranging from 58.1 to 81.6 nmol/L experienced a hazard ratio of 0.28 (95% CI: 0.14–0.55), whereas female participants did not show a significant difference at these levels in comparison to those with serum 25(OH)D concentrations below 58.1 nmol/L. Regarding the ethnic subgroup, higher serum 25(OH)D concentrations corresponded with a greater reduction in cardiovascular mortality among non-Hispanic Whites relative to other ethnicities, characterized by a significant decrease in HRs in accordance with increasing serum 25(OH)D concentrations, and this trend was not observed in other ethnicities subgroup. While other outcomes did not show significant different in HR among ethnicity subgroups.

**Table 4:** Subgroup analysis on the relationship between serum 25(OH)D concentrations and the risk of all-cause, cardiovascular, and cancer-specific mortality in cancer survivors

Subgroup	Serum 25(OH)VD concentration (nmol/mL)			*P for linear trend*	*P for interaction*
	T1 (< 58.1)	T2 (58.1—81.6)	T3 (> 81.6)		
Gender	All-cause mortality				
Males	1.00	0.81 (0.61—1.08)	**0.66 (0.49—0.88)**	0.005	0.27
Females	1.00	0.95 (0.66—1.38)	0.80 (0.56 -1.05)	0.234	
Ethnicity					
Non-Hispanic White	1.00	0.88 (0.69—1.12)	**0.71 (0.56 -0.91)**	0.006	0.48
Other Ethnicities	1.00	**0.55(0.35—0.87)**	0.66(0.36—1.19)	0.067	
Gender	Cardiovascular mortality				
Males	1.00	0.93 (0.54—1.62)	**0.39 (0.21—0.76)**	0.005	0.095
Females	1.00	0.73 (0.39—1.40)	0.60 (0.30—1.22)	0.236	
Ethnicity					
Non-Hispanic White	1.00	**0.80 (0.50—1.28)**	**0.50 ( 0.30—0.84)**	0.014	0.51
Other Ethnicities	1.00	1.02(0.36—2.89)	0.89 (0.34—2.36)	0.84	
Gender	Cancer-specific mortality				
Males	1.00	**0.28 (0.14—0.55)**	0.65 (0.37—1.11)	0.118	0.009
Females	1.00	1.03 (0.53—2.02)	0.77 (0.42—1.42)	0.378	
Ethnicity					
Non-Hispanic White	1.00	0.64 (0.35—1.16)	0.65 (0.42—1.00)	0.07	0.64
Other Ethnicities	1.00	0.65 (0.32—1.31)	0.93 (0.39—2.23)	0.65	

Bold font indicates *p* < 0.05

All models have been adjusted for age, race, gender, marital status, timing of blood collection, *BMI* Body Mass Index, *CCI* Charlson Comorbidity Index, *HEI* Healthy Eating Index, medication usage, smoking status, alcohol consumption, and physical activity. Adjustments exclude the factors used for stratification in the analysis

### Dose–response relationship between serum 25(OH)D concentration with HRs of cause-specific mortality

**Fig. 2:**
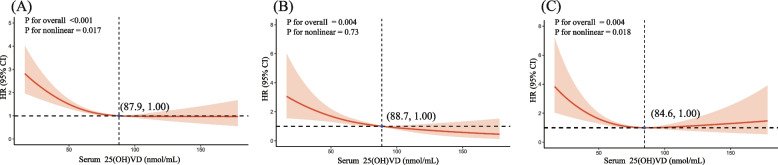
The dose–response curves of the relationship between serum 25(OH) D concentrations and the HRs for all-cause (**A**), cardiovascular (**B**), and cancer-specific (**C**) mortalities. The ribbons indicate the 95% CIs. All models were adjusted by age, gender, ethnicity, marital status, timing of blood collection, BMI, CCI, HEI-2015, smoke status, alcohol consumption, physical activity, cancer types, and time after cancer diagnosed

The RCS models were employed to elucidate the dose–response relationship between serum 25(OH)D concentrations and cause-specific mortality. As illustrated in Fig. 2, significant associations were observed between serum 25(OH)D concentrations and all-cause, cardiovascular, and cancer-specific mortality across all models, HRs and 95% CIs for all-causes, cardiovascular, and cancer-specific mortalities decreased progressively with increasing serum 25(OH)D concentrations up to 87.9 nmol/L, 88.7 nmol/L, and 84.6 nmol/L, respectively, beyond which the HRs plateaued. The RCS curves demonstrated an “L-shaped” configuration for all-cause and cancer-specific mortalities, in contrast, cardiovascular mortality did not exhibit a non-linear relationship with serum 25(OH)D concentrations.

## Discussion

This nationwide prospective study revealed an association between serum 25-hydroxyvitamin D (25(OH)D) levels and mortality among cancer patients. Our results indicate that higher serum 25(OH)D concentrations in cancer survivors correlate with a significant reduction in the risk of all-cause, cardiovascular, and cancer-specific mortality. Additionally, we observed that serum 25(OH)D concentrations exhibited an “L-shaped” relationship with both all-cause and cancer-specific mortality, in contrast to the more linear relationship with cardiovascular mortality. We also estimated the optimal threshold values to minimize mortality risks as 87.9 nmol/L for all-cause, 88.7 nmol/L for cardiovascular, and 84.6 nmol/L for cancer-specific mortality.

Recent studies have explored the association between serum 25(OH)D concentrations and mortality in cancer patients [28–30], suggesting that lower levels are linked to an increased risk of death across different cancer types. Nonetheless, these investigations have overlooked the significance of cause-specific mortality and the identification of an ideal serum 25(OH)D concentration for enhancing the health of cancer survivors. Unlike the general population, cancer survivors face elevated risks of cardiovascular injury [31], endocrine disorders [32], and compromised immune function [33], which are consequences of their treatment. Moreover, they are subject to functional limitations following surgical resection [34, 35], as well as increased levels of stress [36], depression, and the possibility of secondary cancers [37, 38], contributing to a spike in cancer-specific mortality [39]. Therefore, their recommended serum 25(OH)D concentrations may differ from the general population. Our research enriched the field by incorporating cause-specific mortality and characterizing a dose–response curve that estimated the optimal serum 25(OH)D threshold for reducing mortality among cancer survivors.

In this study, we assessed the association between serum 25(OH)D concentrations and primary cause-specific mortality in cancer survivors. Consistent with previous studies, our findings confirm that higher levels of serum 25(OH)D correlate with a reduced mortality risk. This association’s robustness is underscored by various sensitivity and stratified analyses. Additionally, our study reveals new insights. Firstly, subgroup analysis indicated that higher serum 25(OH)D concentrations significantly reduced mortality risk in males, exhibiting a notable interaction effect between gender and serum 25(OH)D concentration in cancer-specific mortality. This contrasts with earlier studies involving other populations [40, 41], where no such interaction was observed, suggesting that vitamin D plays a particularly crucial role in the health of male cancer survivors. Future research should delve into the mechanisms underlying this phenomenon. Secondly, the dose–response relationship between serum 25(OH)D concentrations and cardiovascular mortality risk was more linear, whereas relationships with all-cause and cancer-specific mortality were markedly “L-shaped”. This implies distinct effects of vitamin D on cardiovascular versus cancer development. It should also be noted that due to reduced sample sizes in the subgroup analyses and the limited range of participants with high serum 25(OH)D concentrations, our results must be approached with caution and warrant further validation in larger cohorts.

Furthermore, we determined the serum 25(OH)D concentration thresholds that correlate with the lowest risk of cause-specific mortality. Remarkably, these thresholds exceed the serum 25(OH)D concentrations presently advised for the general population, suggesting that our findings could potentially inform revisions to nutritional recommendations for cancer survivors. Studies encompassing diverse cohorts, such as individuals with hypertension [11], type 2 diabetes [12, 41], hyperlipidemia [13], metabolic dysfunction-related fatty liver disease [40], postmenopausal women [42], and patients with psoriasis [43], has similarly elucidated the dose–response relationship between serum 25(OH)D concentration and mortality risk. Notably, their RCS curves indicated the optimal lowest serum 25(OH)D concentrations were below the commonly recommended concentration of 75 nmol/L. Table 5 succinctly compiles studies that have determined serum 25(OH)D thresholds for the lowest adverse outcome risks. Therefore, to estimate the optimal serum 25(OH)D concentration, and conducted personal recommendation of VD monitor for specific population is necessary.

**Table 5:** Optimal serum 25(OH)D threshold values for minimizing the risk of adverse outcomes in diverse populations as previously reported

Population	Threshold value (nmol/L)	Outcomes
**Osteoarthritis** [44]	27.7	Cardiovascular mortality
	54.4	All-cause mortality
**Hyperlipidemia** [13]	63	Cardiovascular mortality
	64	All-cause mortality
**Type 2 Diabetes**	50	Dementia [45]
	50	Cardiovascular disease [46]
**Adolescents** [47]	58.1	Refractive status
**Coronary Heart Disease** [48]	50	Recurrent Cardiovascular Events
**Metabolic dysfunction associated fatty liver disease** [40]	50	All-cause mortality
**Australian community** [49]	55	Cardiovascular mortality
	65	All-cause mortality
**Dutch population** [50]	46—68	Multiple outcomes (mortality, hypertension, cardiovascular disease, etc.)
**COVID-19** [51]	41.19	Infection and Severity
**Cancer survivors (the present study)**	87.9	All-cause mortality
	88.7	Cardiovascular mortality
	84.6	Cancer-specific mortality

From a cell molecular biology perspective, VD plays a crucial role in modulating the functional dynamics of cancer cells. The expression of the vitamin D receptor (VDR) is noted across various cancer cell types [52], and an extensive array of studies has shown that 1,25-dihydroxyvitamin D3 and its derivatives can inhibit cancer cell proliferation, promote apoptosis, interfere with angiogenesis, and modify cell adhesion and migration, thereby attenuating the invasiveness of cancer cells [53–55]. These actions hinge on the presence of the VDR. Adequate VD levels in cancer survivors may bolster the effectiveness of cancer therapies [56], safeguard cardiovascular health [57], and modulate both adaptive and innate immune responses [2], thus potentially enhancing their survival prospects. Moreover, given the observed decline in the expression of CYP27B1, VDR, CYP11A1, and RORα/γ during cancer progression, as previously discussed, the utility of VD-based treatments may be confined to early rather than advanced stages of the disease [30]. Consequently, vigilant monitoring and maintenance of VD concentrations in cancer survivors, particularly those in the early stages, could serve as a strategic approach to promote better long-term outcomes.

The primary strength of this study is the inclusion of a large population with long observation period. Data derived from the NHANES survey enabled us to adjust the models to account for variables such as participants' baseline characteristics, dietary habits, comorbidities, and lifestyle factors. Nonetheless, although serum 25-hydroxy VD (25(OH)D) is a reliable biomarker typically indicative of VD status for approximately two months, these levels may fluctuate over time. Nevertheless, in this study, serum 25(OH)D concentrations were only measured at a single time point, which may not reflect the long-term status. Moreover, various cancer types, stages, and treatment approaches can impact the anticipated survival duration. For more accurate results, these factors were expected to be accounted for in the future study. Nevertheless, the cancer survivor participants recorded in NHANES predominantly exhibited favorable survival prognoses as many research reported before [58, 59], and we have excluded patients who likely suffered from advanced-stage cancer, which represented only a minority of cases, and most of participants included in the present study exhibited long survival periods for more than 5 years after cancer diagnosed. Consequently, the survival bias in the current study remains manageable. Furthermore, another limitation in the present study is that dietary patterns [60], sun exposure [61], and genetic diversity vary globally [62], and as such, it is not possible to simply generalize our findings to other populations.

While the limitations certainly exist, the study also offers novel insights into the prognostic assessment of cancer survivors. The findings suggest that optimal serum 25(OH)D concentrations for cancer survivors differ significantly from those of the general population, and they require a higher serum 25(OH)D concentration to reduce mortality risk.

## Conclusion

The current study reveals a significant association between serum 25(OH)D concentration and all-cause, cardiovascular, and cancer-specific mortality among cancer survivors. Incremental increases in serum 25(OH)D concentrations were linked to diminishing risks up to thresholds of 87.9 nmol/L for all-cause mortality, 88.7 nmol/L for cardiovascular mortality, and 84.6 nmol/L for cancer-specific mortality. These threshold values surpass the typically suggested concentration of 75 nmol/L, suggesting that cancer survivors may need higher doses of vitamin D.

### Supplementary Information

**Supplementary Material 1.**

## Data Availability

The data that support the findings of this study are available from NHANES. Data are publicly available at the National Center for Health Statistics of the Center for Disease Control and Prevention, the URL is as followed, https://wwwn.cdc.gov/nchs/nhanes/Default.aspx
