# Supplementary material for: Association of serum 25-hydroxy-vitamin D concentration and risk of mortality in cancer survivors in the United States
Source: BMC Cancer. 2024 Apr 30;24:545. doi: 10.1186/s12885-024-12304-8 (PMC11061943; doi:10.1186/s12885-024-12304-8)
Supplement: Supplementary file 1 — Supplementary Material 1. [file 12885_2024_12304_MOESM1_ESM.docx]

1. Correlations among variables in the present study

Variables in the present study were estimated their correlation by Spearmen correlation coefficient. As figure 2 shown, variables represented weak correlation by each other with the coefficients between -0.25 - 0.22. Then, variance inflation factors of the variables were calculated and with the results of 1.12 [25(OH)D], 1.18 (gender), 1.34 (age), 1.13 (race), 1.07 (marital status), 1.08 (BMI), 1.13 (CCI), 1.13 (medication usage), 1.08 (HEI), 1.08 (smoke status), 1.08 (alcohol drinking), 1.09 (cancer type), 1.04 (MET value), and 1.04 (blood collection period) respectively. Therefore, the variables in the present study could be treated as independent and did not exist multicollinearity problem.


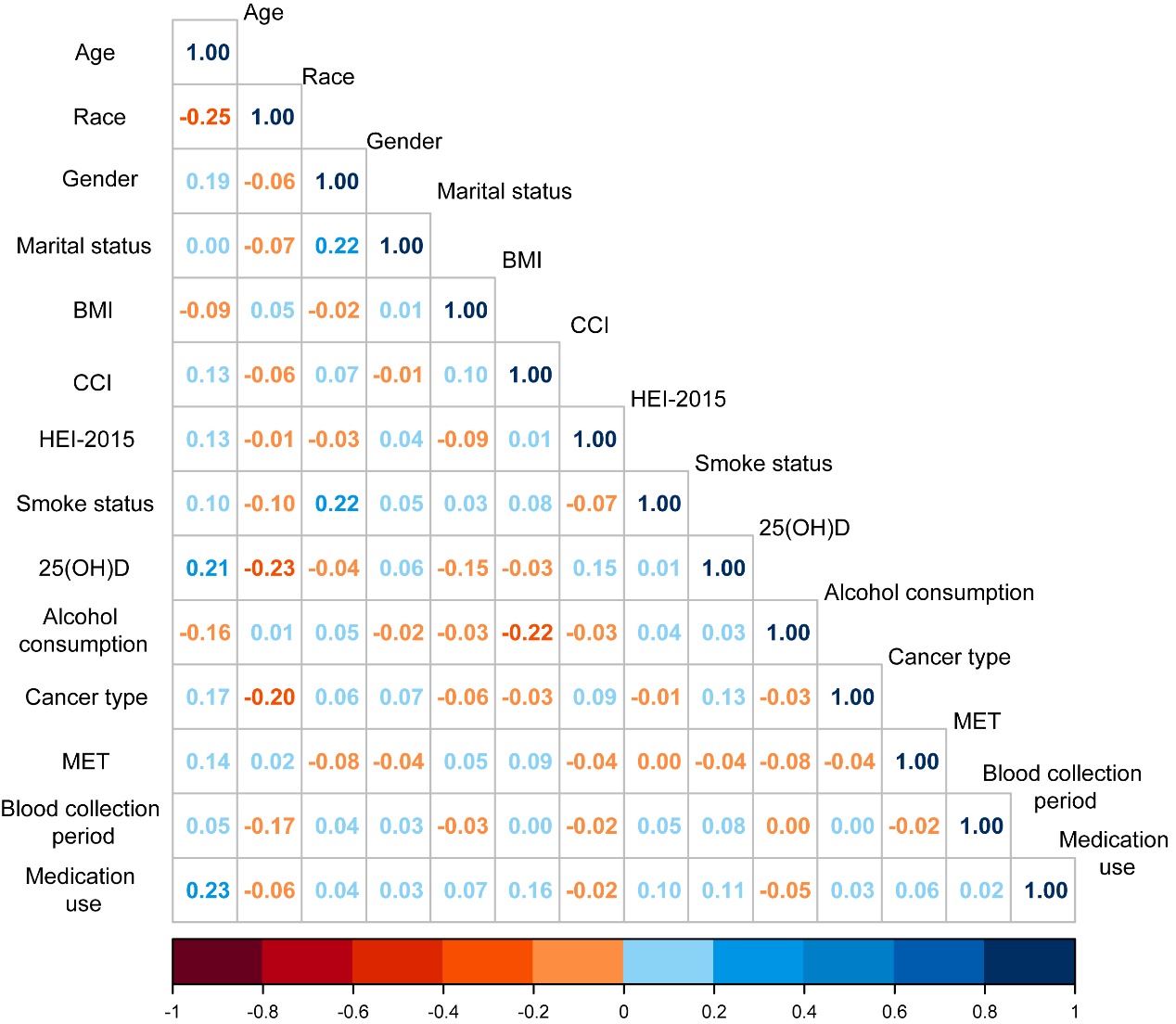


Figure 1. The Spearmen correlation coefficients among variables in the present study

2. Schoenfeld residuals tests for the COX proportional hazard models

Schoenfeld residuals tests were conducted for the Cox proportional hazard models. As shown in Figure 2, the Schoenfeld individual test for the main variable, serum 25(OH)D concentration, yielded a p-value greater than 0.05. Furthermore, the global Schoenfeld test for the multivariable Cox models also resulted in a p-value exceeding 0.05, indicating that the models meet the assumption of proportional hazards.


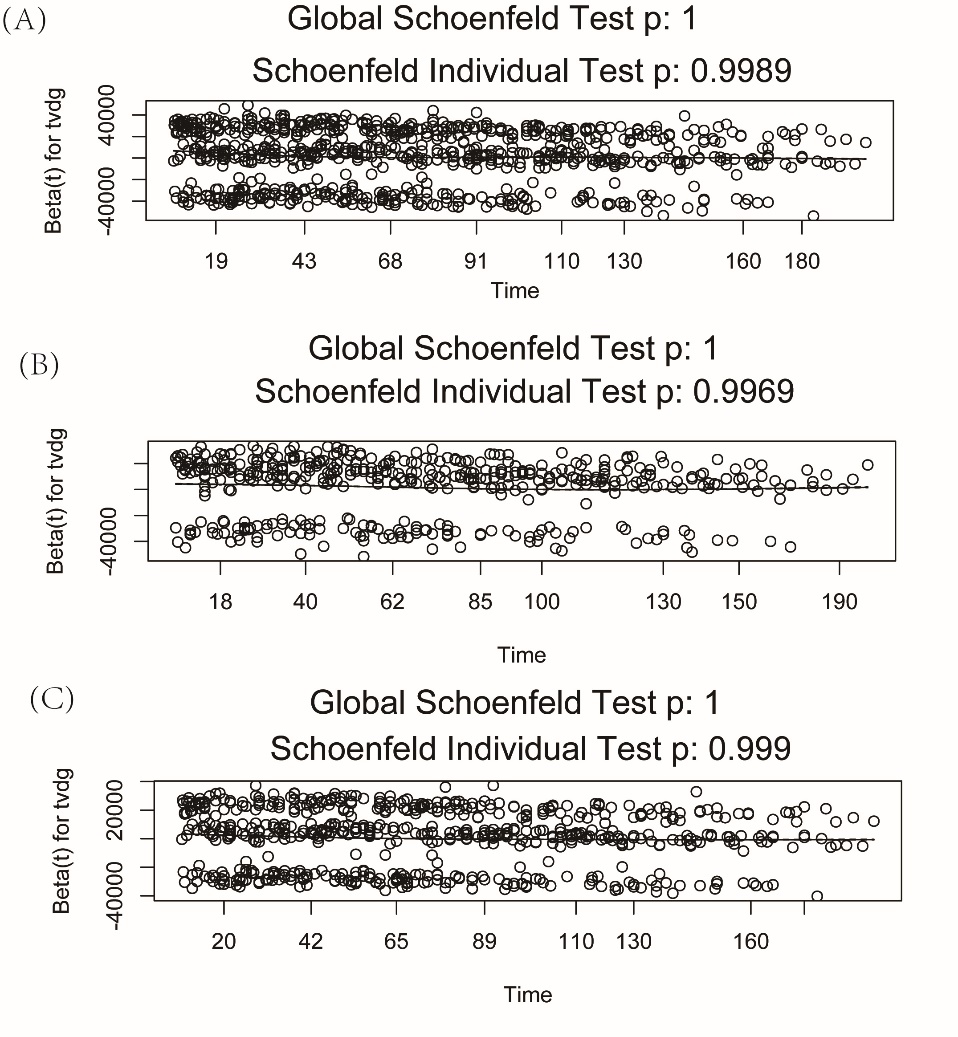


Figure 2. The Schoenfeld residuals tests for the Cox proportional hazard models in the present study: (A) the model encompassing all samples, (B) the model for gender subgroup analysis, and (C) the model for ethnicity subgroup analysis.
